# Nencki Affective Word List (NAWL): the cultural adaptation of the Berlin Affective Word List–Reloaded (BAWL-R) for Polish

**DOI:** 10.3758/s13428-014-0552-1

**Published:** 2015-01-15

**Authors:** Monika Riegel, Małgorzata Wierzba, Marek Wypych, Łukasz Żurawski, Katarzyna Jednoróg, Anna Grabowska, Artur Marchewka

**Affiliations:** 1 Laboratory of Brain Imaging, Neurobiology Centre, Nencki Institute of Experimental Biology, Warsaw, Poland; 2 Laboratory of Psychophysiology, Department of Neurophysiology, Nencki Institute of Experimental Biology, Warsaw, Poland; 3 University of Social Sciences and Humanities, Warsaw, Poland

**Keywords:** Emotion, Affective verbal stimuli, Affective ratings, Valence, Arousal, Imageability, Berlin affective word list–reloaded, Nencki affective word list

## Abstract

In the present article, we introduce the Nencki Affective Word List (NAWL), created in order to provide researchers with a database of 2,902 Polish words, including nouns, verbs, and adjectives, with ratings of emotional valence, arousal, and imageability. Measures of several objective psycholinguistic features of the words (frequency, grammatical class, and number of letters) are also controlled. The database is a Polish adaptation of the Berlin Affective Word List–Reloaded (BAWL-R; Võ et al., *Behavior Research Methods 41*:534–538, 2009), commonly used to investigate the affective properties of German words. Affective normative ratings were collected from 266 Polish participants (136 women and 130 men). The emotional ratings and psycholinguistic indexes provided by NAWL can be used by researchers to better control the verbal materials they apply and to adjust them to specific experimental questions or issues of interest. The NAWL is freely accessible to the scientific community for noncommercial use as supplementary material to this article.

**Electronic supplementary material:**

The online version of this article (doi:10.3758/s13428-014-0552-1) contains supplementary material, which is available to authorized users.

As was indicated by Wierzbicka (1999), human beings are “classifying animals” that categorize the “contents of the world” and events into categories and put labels on them. One of the things that undergo categorization is feelings, and the labels do not match across language boundaries.

Being omnipresent in everyday life and directly available for experimental use, words belong to the most widely used stimuli in cognitive psychology, affective, and cognitive neuroscience, as well as in neighboring disciplines. Using word stimuli, as compared with using visual (Lang, Bradley, & Cuthbert, 2008; Marchewka, Żurawski, Jednoróg, & Grabowska, 2014) and auditory (Belin, Fillion-Bilodeau, & Gosselin, 2008; Bradley & Lang, 1999) stimuli, has numerous advantages, the greatest of which is that they can be strictly controlled for physical attributes and linguistic variables such as frequency of use, familiarity, word length, similarity to other words, word onset, age of acquisition, and the imagery and concreteness of the underlying concept. It is known that linguistic variables of this kind significantly influence the processing of verbal material (Moors et al., 2013; Võ, Conrad, Kuchinke, Urton, Hofmann, & Jacobs, 2009; Võ, Jacobs, & Conrad, 2006). Another advantage is that verbal stimuli are graphically less complex and less variable with regard to their physical features than are the majority of picture stimuli, which need to be controlled for complexity, brightness, color, and contrast (Soares, Comesaña, Pinheiro, Simões, & Frade, 2012) to make them comparable. However, these advantages of using verbal stimuli in studies of emotions cannot be exploited unless normative ratings for their affective content are available.

Currently, a number of databases offer affective norms for words in different languages, summarized in Table 1. Regardless of the increasing interest in this type of material, only recently was the first attempt to create an equivalent instrument for the Polish language made, with emphasis put on the duality-of-mind approach for emotion formation (Imbir, 2014).

**Table 1:** Available affective verbal data sets

Authors	Name	Language	*N* of Words	Grammatical Class	Psycholinguistic Variables	Categories	Emotional Dimensions	Discrete Emotions	Scales	Source(s)
Bradley & Lang, 1999	ANEW	English	1,034	nouns, verbs, adjectives	frequency	–	valence, arousal, dominance	–	SAM	Mehrabian & Russell, 1974; Bellezza, Greenwald, & Banaji, 1986
Altarriba, Bauer, & Benvenuto, 1999	–	English	326	nouns, verbs, adjectives	concreteness, imageability, context availability	–	–	–	7-point scales (ranging from 1 to 7)	*Dictionary of Affect in Language* (WhisseIl, 1989); previous studies investigating emotions (e.g., Shaver, Schwartz, Kirson, & O’Connor, 1987)
Võ et al., 2006	BAWL	German	2,200	verbs, nouns	imageability	–	valence	–	7-point scales (ranging from –3 to 3, 1 to 7)	CELEX database (Baayen et al., 1993)
Stevenson, Mikels, & James, 2007	–	English	1,034	nouns, verbs, adjectives	–	–	–	happiness, anger, sadness, fear, disgust	5-point scales (ranging from 1 to 5)	ANEW
Redondo et al., 2007	–	Spanish	1,035	nouns, verbs, adjectives	number of letters, syllables, orthographic neighbors, grammatical class, frequency, familiarity, concreteness, imageability	–	valence, arousal, dominance	–	SAM	ANEW translation
Janschewitz, 2008	-	English	460	nouns, verbs, adjectives	personal use, offensiveness, familiarity, tabooness, imageability	–	valence, arousal	–	9-point scales (ranging from 1 to 9)	Jay, 1992; ANEW
Võ et al., 2009	BAWL-R	German	2,900	nouns, verbs, adjectives	imageability, number of letters, syllables, phonemes, orthographic neighbors frequency, bigram frequency, accent	–	valence, arousal	–	7-point scales (ranging from –3 to 3 and 1 to 7), SAM	CELEX database (Baayen et al., 1993)
Lahl, Göritz, Pietrowsky, & Rosenberg, 2009	–	German	2,654	nouns	imageability, meaningfulness, potency, number of letters, frequency	–	valence, arousal	–	11-point scales	electronic corpus of the Institute of German Language and Linguistics of the Humboldt University of Berlin
Kanske & Kotz, 2010	LANG	German	1,000	nouns	concreteness	–	valence, arousal	–	9-point scales (SAM, concrete-abstract)	previously rated word list (Kanske & Kotz, 2007); dictionary (Duden: Die deutsche Rechtschreibung, 2000)
Eilola & Havelka, 2010		English/ Finnish	210	nouns	offensiveness, concreteness, familiarity	taboo words	valence, emotional charge	–	fully sliding visual analogue scales	ANEW and taboo words for which the American English norms were collected by Janschewitz, 2008
Briesemeister, Kuchinke, & Jacobs, 2011	DENN-BAWL	German	1,958	nouns	–	–	–	happiness, anger, sadness, fear, disgust	5-point scales (ranging from 1 to 5)	BAWL-R
Stevenson et al., 2011	ISAWS	English	1,450	–	sexual valence, arousal, energy	sexual words	valence, arousal, dominance	happiness, anger, sadness, fear, disgust	9-point scales (ranging from 1 to 9; SAM)	ANEW (Bradley & Lang, 1999; Stevenson et al., 2007), others
Ferré et al., 2012	–	Spanish	380	nouns	concreteness, familiarity, frequency, number of letters	animals, people, objects	valence, arousal	–	9-point scales (ranging from 1 to 9; SAM, numerical scale)	ANEW translation; the database of Pérez Dueñas et al., 2010; the Redondo et al., 2005, normative data
Soares et al., 2012	–	Portuguese	1,034	nouns, verbs, adjectives	–	–	valence, arousal, dominance	–	SAM	ANEW translation
Gilet, Grühn, Studer, & Labouvie-Vief, 2012	FEEL	French	835	adjectives	imagery	–	valence, arousal	–	7-point scales	web-based database of French words (LEXIQUE; New, Pallier, Ferrand, & Matos, 2001); previous French rating studies (Flieller & Tournois, 1996; Niedenthal et al., 2004)
Moors et al., 2013	–	Dutch	4,300	nouns, verbs, adjectives, adverbs	age of acquisition	–	valence, arousal, dominance	–	7-point scales	De Deyne & Storms, 2008; Fontaine, Poortinga, Setiadi, & Suprapti, 2002; Fontaine et al., 2007; Frijda, Kuipers, & ter Schure, 1989; Hermans & De Houwer, 1994; Keuleers, Diependaele, & Brysbaert, 2010; Osgood et al., 1957; Rouckhout & Schacht, 2000; http://synoniemen.net/
Ric, Alexopoulos, Muller, & Aubé, 2013	–	French	524	adjectives	consequences for the possessor of the trait, consequences for the others interacting with the trait holder	–	valence	happiness, anger, sadness, fear, disgust	7-point scales	previous research (Anderson, 1968; Boies, Lee, Ashton, Pascal, & Nicol, 2001; Wentura et al., 2000) and brainstorming
Söderholm et al., 2013	–	Finnish	420	nouns	number of letters, surface frequency, lemma frequency, bigram frequency, initial trigram frequency, final trigram frequency	–	valence, arousal	–	7-point scale (ranging from 1 to 7)	ANEW translation; the Turun Sanomat corpus
Warriner et al., 2013	–	English	13,915	nouns, verbs, adjectives	age of acquisition, frequency, imageability, sensory experience	taboo words and other categories	valence, arousal, dominance	–	9-point scales (ranging from 1 to 9)	ANEW; Van Overschelde, Rawson, and Dunlosky’s, 2004, category norms, and the SUBTLEX-US corpus (Brysbaert & New, 2009)
Montefinese et al., 2014	–	Italian	1,121	nouns, verbs, adjectives	familiarity, imageability, concreteness	–	valence, arousal, dominance	–	9-point scales (ranging from 1 to 9)	ANEW translation; semantic norms collected in the authors' laboratory (Montefinese et al., 2013)
Quadflieg et al., 2014	–	French	875	adjectives	applicability to humans, applicability to nonhuman entities, concreteness, familiarity, intensity, temporal stability, visibility, grammatical class, number of letters, number of syllables, frequency	–	valence	–	7-point scales	various French dictionaries, as well as previous French rating studies (e.g., Boies et al., 2001; Gilet et al., 2012; Niedenthal et al., 2004)
Monnier & Syssau, 2014	FAN	French	1,031	nouns, adjectives	number of letters, number of phonemes, number of syllables, grammatical class, frequency, imageability	objects, animals, food, concepts	valence, arousal	–	SAM	affective database compiled by Syssau and Font, 2005; affective database compiled by Bonin et al., 2003; the French Lexique database (New et al., 2004)
Schmidtke et al., 2014	ANGST	German	1,003	nouns, verbs, adjectives	imageability, potency, frequency, grammatical class, number of letters, number of syllables, orthographic neighbors	–	valence, arousal, dominance	–	7-point scales (ranging from –3 to 3, 1 to 7); SAM	ANEW translation
Imbir, 2014	ANPW	Polish	1,584	nouns, verbs, adjectives, adverbs, two-word phrases	part of speech, number of letters, frequency	–	valence, arousal, dominance, origin, significance, source	–	9-point scales (ranging from 1 to 9); SAM	ANEW translation, own research

The Nencki Affective Word List (NAWL) was constructed using a dimensional view of emotions, which assumes that emotion can be defined as the coincidence of values on a number of different dimensions. This view was first described in Osgood’s (Osgood, Suci, & Tannenbaum, 1957) seminal work *The Measurement of Meaning.* He described a semantic differential in which factor analyses conducted on a wide variety of verbal judgments indicated that the variance in emotional assessments was accounted for by three major dimensions. The two primary dimensions were affective *valence*, ranging from pleasant to unpleasant, and *arousal*, ranging from calm to excited. A third, less strongly related dimension was *dominance*, or in other words, control. The two primary dimensions were selected to be used in the construction of the NAWL. Instead of the third one, imageability was chosen, following the dimensions distinguished by the Berlin Affective Word List–Reloaded (BAWL-R; Võ et al., 2009). This decision was motivated by the fact that a number of studies investigating the effects of emotional valence on word memory have not controlled for the imageability of words, in spite of the evidence that easily imageable words are processed more efficiently and memorized better than words that are more difficult to imagine (Nittono, Suehiro, & Hori, 2002).

Taking into consideration the constantly growing number of behavioral and neuroimaging studies on emotion, conducted on both cross-cultural and Polish populations, we have provided the NAWL, containing 2,902 Polish words with subjective ratings for emotional valence, emotional arousal, and imageability, along with data on several psycholinguistic factors known to influence word perception. This newly developed data set is freely available to the scientific community for noncommercial use as supplementary material to this article.

## Method

### Materials

The words included in the NAWL are basically Polish translations of the items from BAWL-R (Võ et al., 2009), which contains 2,902 German words selected from the CELEX database (Baayen, Piepenbrock, & Van Rijn, 1993) and the original BAWL (Võ et al., 2006). All of the words are emotionally loaded and are characterized by negative, neutral, or positive affective valence.

The translation of the original German list of words was performed according to the rules described below and was subjected to further cultural modifications. Initially, four independent translators proficient in the German language were asked to translate the list very carefully and to find the best (defined as the most prototypical, most often used, and most emotional) equivalents in the Polish language. Subsequently, the translations were checked for coherence. Most of the words were identical in all four translations. In some cases, three of the four translators were unanimous (75 % agreement), two of the our translators (50 % agreement) were unanimous, or none of the four were unanimous (0 agreement). Those were the cases for 697, 273, and 24 words, respectively. In these cases, the translators were asked to review their translations, give more synonyms, and assess other possible versions of the translation. For instance, the German word *Ganove* was translated as *bandzior* (Eng. “mugger”), *oprych* (“thug”), *rzezimieszek* (“raider”), and *oszust* (“swindler”).

Once agreement was reached between the translators and the proper versions of translations were chosen for all 2,902 words, two other, independent translators proficient in the German language were asked to translate the list back from Polish to German, in order to check the validity of the translations. They were asked to make the translations very carefully and to find the best (most prototypical, most often used, and most emotional) equivalents in the German language. In most cases, the resulting back-translations were the same words from the BAWL-R. In some cases (*n* = 201), none of the back-translators matched the original word, or in others only one of the translators did (280 for the first translator, 37 for the second). In these cases, the translators were asked to compare their translations with the original words from the list and decide whether they would translate the words this way or if they found the Polish translation improper. All changes introduced at this stage were done in consultation with the other four translators.

In the list of 2,902 Polish affective words obtained in the process of translation and back-translation, 195 words were found to have duplicates. These words were replaced with other words: either different translations proposed by some of the translators or synonyms found in the *Polish Dictionary of Synonyms* (Broniarek, 2006).

Attention was also paid to preserve the grammatical structure of Polish language—that is, the proper proportion of nouns, verbs, and adjectives. One of the most important dictionaries that provides an overall analysis of the grammatical structure of the Polish language is the *Polish Language Dictionary*, edited by Doroszewski in 1958-69. Although this dictionary is relatively old, according to Bańko (1992) the grammatical structure is rather stable, and the data provided by this dictionary are also representative for contemporary Polish: nouns, 54.9 %; verbs, 19.5 %; adjectives, 19.6 %; adverbs, 3.8 %; and other words, 2.33 %. This percentages of nouns, verbs, and adjectives were applied to the NAWL.

Another important psycholinguistic and lexical property of the words is their frequency of use per million words in Polish. This was retrieved for all of the words from the list, with the use of the balanced National Corpus of Polish Language, containing over 290 million word segments (NKJP; Pęzik, 2012). All words with a frequency lower than 0.003 per million were replaced with other, more frequent synonyms.

In addition to the norms of frequency mentioned above, we included recently published Polish word frequencies based on movie subtitles, which thus better reflect the spoken language (SUBTLEX-PL; Mandera, Keuleers, Wodniecka, & Brysbaert, 2014). Apart from the raw frequencies for the word forms, SUBTLEX-PL offers frequencies transformed to the Zipf logarithmic scale, measures of contextual diversity, part-of-speech-specific word frequencies, and frequencies of associated lemmas and word bigrams, providing researchers with necessary tools for conducting psycholinguistic research in Polish.

Some of the items from the original Berlin Affective Word List were culturally specific for the German society—for instance, were connected with specific geographical names or constituted stereotypes about some social groups. These items were replaced with items specific to the Polish language and society. For instance, *Prater*—a name of a big park in Vienna—was replaced with the name of a Warsaw park—*Łazienki*.

The final version of the NAWL consists of 2,902 words and includes 1,676 nouns, 614 verbs, and 612 adjectives, which account for 54.3 %. 19.9 %, and 19.8 %, respectively, of the total word number. Each word is represented only once, and each contains 2–17 letters (mean [*M*] = 7.37; standard deviation [*SD*] = 2.35). The frequencies of the words (as measured in everyday language use) range from 0.0034 to 15,793 (*M* = 55.1, *SD* = 321.2). As for the proportions of grammatical classes, these are very close to the general proportions of grammatical classes in Polish.

### Participants

A total of 266 healthy volunteers (130 male, 136 female) from the ages of 20–52 (*M* = 23.7 years, *SD* = 4.9) took part in the study. The participants were of Polish origin, and all of them were proficient speakers of Polish. They were mainly college students and young employees living in Warsaw. They were educated in the following fields: arts (m = 39, *f* = 40), mathematics (m = 37, f = 29), engineering (m = 19, f = 9), biological and psychological sciences (m = 26, f = 48), medicine (m = 0, f = 5), mixed (m = 3, f = 2), or no higher education (m = 6, f = 3). The local Research Ethics Committee in Warsaw approved the experimental protocol of the study, and written informed consent was obtained from all of the participants prior to the experiment.

The students were invited to participate in the study through advertising posters at several departments of Warsaw universities. Additionally, all of the participants of the initial study sessions were asked to invite their friends to take part in the study. All participants obtained a financial reward of 30 PLN (approximately €7).

### Procedure

Once they had filled in the informed consent form, all of the participants individually completed one of the assessment rounds through a platform available on a local server. Up to 10 people sat in one room during the assessment session, working on separate computers, with an average distance of 60 cm from the screen. Completing the task at their own pace took approximately 30–60 min. All participants signed an obligation not to tell anybody about the details of the experiment until 30 days had passed.

The evaluation procedure was based on the German one, and involved collection of the words’ ratings on the following scales: Valence, Arousal, and Imageability. Details regarding the scales are given in the next section.

Before the assessment session, the participants were given detailed instructions about the stimuli to be presented and the procedure. All participants were informed that should they feel any discomfort during the session, they could report it immediately in order to quit the experiment.

**Fig. 1:**
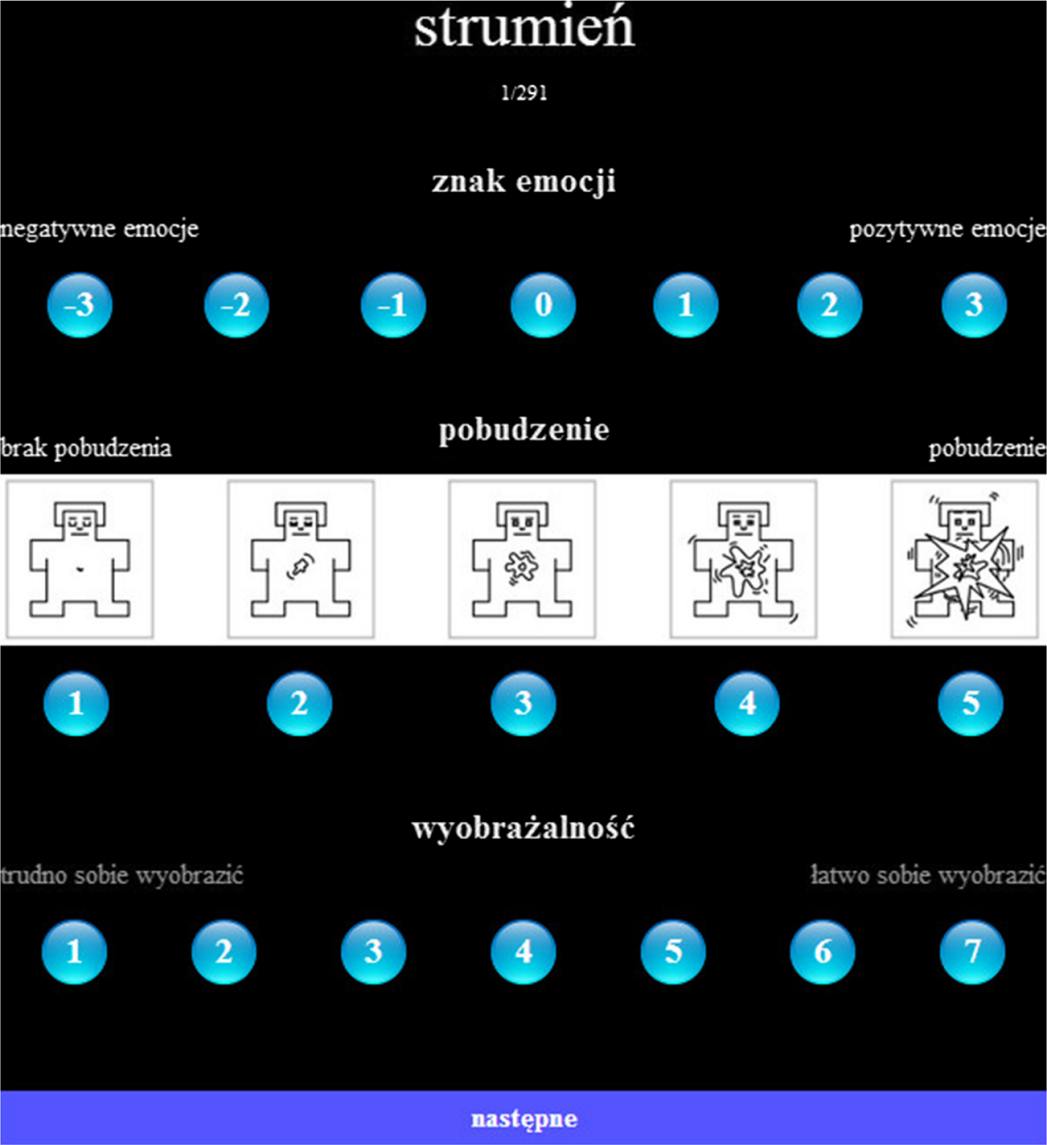
Example of the assessment platform for the first of 291 words, *strumień* (Eng. “stream”). pl. *strumień*, Eng. “stream”; *znak emocji*, “valence”; *negatywne emocje*, “negative emotions”; *pozytywne emocje*, “positive emotions”; *pobudzenie,* “arousal”; *brak pobudzenia*, “no arousal”; *wyobrażalność*, “imageability”; *trudno sobie wyobrazić*, “difficult to imagine”; *łatwo sobie wyobrazić*, “easy to imagine”; *następne*, "next"

During the assessment session, each participant was presented with 291 words chosen pseudorandomly from all the categories, under the following constraints: no more than two words from each affective valence category (positive, neutral, and negative), and no more than four words from each grammatical category occured consecutively. In order to avoid serial-position and recency effects, each subset of 291 words was divided into three parts, and these parts were positioned in three possible ways, counterbalanced between the participants. Single words were presented in full-screen view for 2 s. Each presentation was followed by an exposure of the rating scales (for the assessments of valence, arousal, and imageability) on the new screen, accompanied by the word presented in smaller font in the upper part of the screen (see Fig. 1). The word and rating scales remained available to the participants’ view until they had completed all three ratings. After the participants had completed the ratings, they had to press the “Next” button, which triggered exposure of the next word in the series. A short break was introduced after participants had rated the first half of the words.

### Rating scales

As we mentioned above, all of the words were rated on three continuous scales: emotional valence, arousal, and imageability. The scale of emotional valence was used to estimate how negative or positive were the emotions evoked by a given word, ranging from –3 to 3 (–3 for *very negative emotions*, 0 for *emotionally neutral emotions*, and 3 for *very positive emotions*). On the scale of arousal, participants estimated to what extent a particular word made them feel aroused/excited or unaroused/relaxed, assessed with the use of self-assessment manikins (SAMs; Lang, 1980) ranging from 1 to 5 (1 for *unaroused*, 3 for *neutral*, and 5 for *very much aroused*, e.g., jittery or excited). The authors of BAWL-R (Võ et al., 2009) reported that SAMs were used for depicting increasing levels of arousal in order to circumvent the sexual connotations implied by the German word for “arousal” (*Erregung*). Following the arguments given by Võ et al. (2009), the arousal SAM was used as a 5-point instead of a 9-point scale, because in a pilot arousal-rating study that had included a 9-point scale, with the possibility of marking points between the five depictions, such fine-grained ratings were not used by the participants. The third scale was for ratings of imageability, defined as the ease with which a word gives rise to a sensory mental image, ranging from 1 to 7 (1 = *it is very difficult to imagine what is described by this word*, 7 = *it is very easy to imagine what is described by this word*). The Appendix contains the original instructions in Polish and their English translation. Participants indicated their ratings by clicking the proper button on the screen with a standard computer mouse (see Fig. 1).

## Results

### Ratings of the affective variables

For each word, we obtained from 25 to 54 ratings (*M* = 26.65, *SD* = 1.49) on each scale from the 266 participants of the study. Each word was rated by both males and females, with respective average numbers of ratings of 13.03 (*SD* = 2.26; ranging from 9 to 30) for males, and 13.62 (*SD* = 2.48; ranging from 10 to 33) for females.

In order to provide researchers with a general overview of the content of the database, descriptive statistics for the valence and arousal ratings, along with psycholinguistic indices (including imageability), are presented in Table 2.

**Table 2:** Descriptive statistics calculated separately for each dimension in men, women, and both groups, for all the NAWL words

	Min	Max	*M*	*SD*
Affective dimension/ sex				
Valence/ M	–2.57	2.73	0.20	1.08
Valence/ W	–2.92	2.94	0.14	1.04
Valence/ all	–2.73	2.76	0.17	1.08
Arousal/ M	1.07	4.21	2.41	1.06
Arousal/ W	1.00	4.38	2.37	1.08
Arousal/ all	1.11	4.27	2.38	1.08
Psycholinguistic subj. index/ sex				
Imageability/ M	2.43	7.00	5.59	1.32
Imageability/ W	2.08	7.00	5.56	1.39
Imageability/ all	2.67	6.89	5.60	1.38
Psycholinguistic obj. index				
Number of letters	2	17	7.37	2.35
Frequency	0	15,793	55.19	321.27
Psycholinguistic obj. index		*N*	*V*	*A*
Grammatical class (number of words)		1,676	614	612

*Min* minimal value, *Max* maximal value, *M* mean, *SD* standard deviation, *n* number, *M* men, *W* women, *all* both groups, *N* nouns, *V* verbs, *A* adjectives, *subj.* subjective, *obj.* objective

It may be noted that, in line with most of the previous studies, participants did not receive explicit instructions about ambiguous words having different meanings depending on the context. Thus, such ambiguity may be reflected in the rating variability.

### Reliability

Since the applicability of the collected affective norms for Polish in experimental studies is highly dependent on their reliability, we addressed this issue by applying split-half reliability estimations for the whole sample and for the samples of males and females separately. In accordance with the descriptions of this procedure in literature (Monnier & Syssau, 2014; Montefinese, Ambrosini, Fairfield, & Mammarella, 2014; Moors et al., 2013), the samples were split into halves in order to form two groups, depending on the odd and even entrance ranks of the participants. In the case of the whole sample, the odd and even groups included comparable numbers of males and females (f = 26 % in odds, and f = 26 % in evens). First, mean ratings for each word were calculated in each group. Then,these means were correlated between the two groups within the whole sample, the male sample, and the female sample. All of the correlations were significant (*p* < .001). Finally, the correlations were adjusted using the Spearman–Brown formula. The corrected correlations were especially strong for the valence scale (*r* = .97 in the whole sample, *r* = .90 for males, and *r* = .94 for females). We also found strong correlations on the scale of arousal: namely, *r* = .81 for the whole sample, *r* = .67 for males, and *r* = .67 for females. On the scale of imageability, the correlations were as follows: *r* = .87 in the whole sample, *r* = .71 for males, and *r* = .77 for females.

### Correlations between variables

**Fig. 2:**
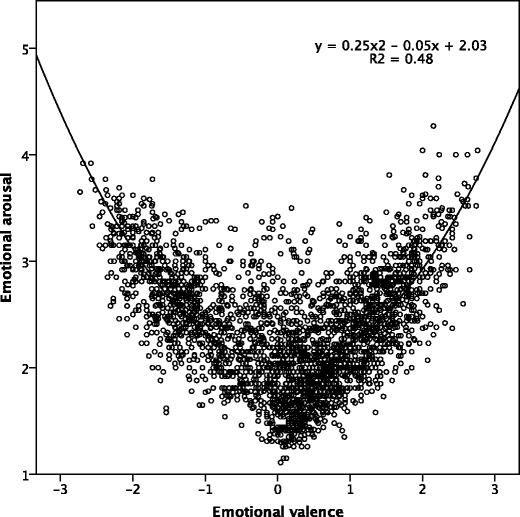
Quadratic and linear functions fitting arousal to the whole range of valence in the Nencki Affective Word List

Figure 2 depicts a function relating emotional arousal and emotional valence in the affective space. This relation can be described by the quadratic function *y* = 0.25*x* ^2^ – 0.05*x* + 2.03, *R* ^2^ = .48. The U-shaped (boomerang-like) function reflects higher arousal values for emotionally valenced words. A boomerang-shaped distribution has been also reported in the studies for other languages (Bradley & Lang, 1999; Eilola & Havelka, 2010; Ferré, Guasch, Moldovan, & Sánchez-Casas, 2012; Kanske & Kotz, 2010; Montefinese et al., 2014; Moors et al., 2013; Redondo, Fraga, Padrón, & Comesaña, 2007; Schmidtke, Schröder, Jacobs, & Conrad, 2014; Soares et al., 2012; Söderholm, Häyry, Laine, Karrasch, & Ha, 2013; Võ et al., 2009; Warriner, Kuperman, & Brysbaert, 2013), as well as for the visual and auditory stimulus materials (Kanske & Kotz, 2011; Lang et al., 2008; Marchewka et al., 2014).

Although NAWL does not contain taboo words, or sexual words such as “orgasm,” which are usually rated as both highly arousing and emotionally positive (Redondo et al., 2007), we found a symmetrical distribution of the ratings on both dimensions in our sample. In other words, both the negative and positive words showed higher arousal ratings than the neutral words. An increasing degree of negative valence was accompanied by an increase in emotional arousal (*y* = 0.13*x* ^2^ – 0.22*x* + 2.08, *R* ^2^ = .46), and this relation held true also for positive valence (*y* = 0.15*x* ^2^ – 0.25*x* + 1.85, *R* ^2^ = .49).

With regard to the relation between these two emotional dimensions and the psycholinguistic subjectively rated index of imageability, no significant linear correlation was found between arousal and imageability. This relation was also best explained by a quadratic function, *y* = 0.22*x* ^2^ – 1.01*x* + 6.72, *R* ^2^ = .01, which means that this function explained only 1 % of the imageability ratings in terms of the ratings of arousal. There was a significant linear correlation between the ratings of valence and imageability, yet the quadratic function *y* = 0.05*x* ^2^ + 0.13*x* + 5.51, *R* ^2^ = .06 gave the best explanation of this relation.

### The impact of sex on emotional evaluations

Sex differences have been found to be a significant issue in neuroscience and psychological research (Cahill, 2006). As far as emotion processing is concerned, different levels of valence and arousal elicited by the same visual stimuli were found in men and women. Specifically, women react more strongly to unpleasant materials, as compared to men, whereas men tend to rate pleasant pictures as being more pleasant and arousing than women do (Bradley, Codispoti, Sabatinelli, & Lang, 2001; Lithari et al., 2010; Wrase et al., 2003).

**Fig. 3:**
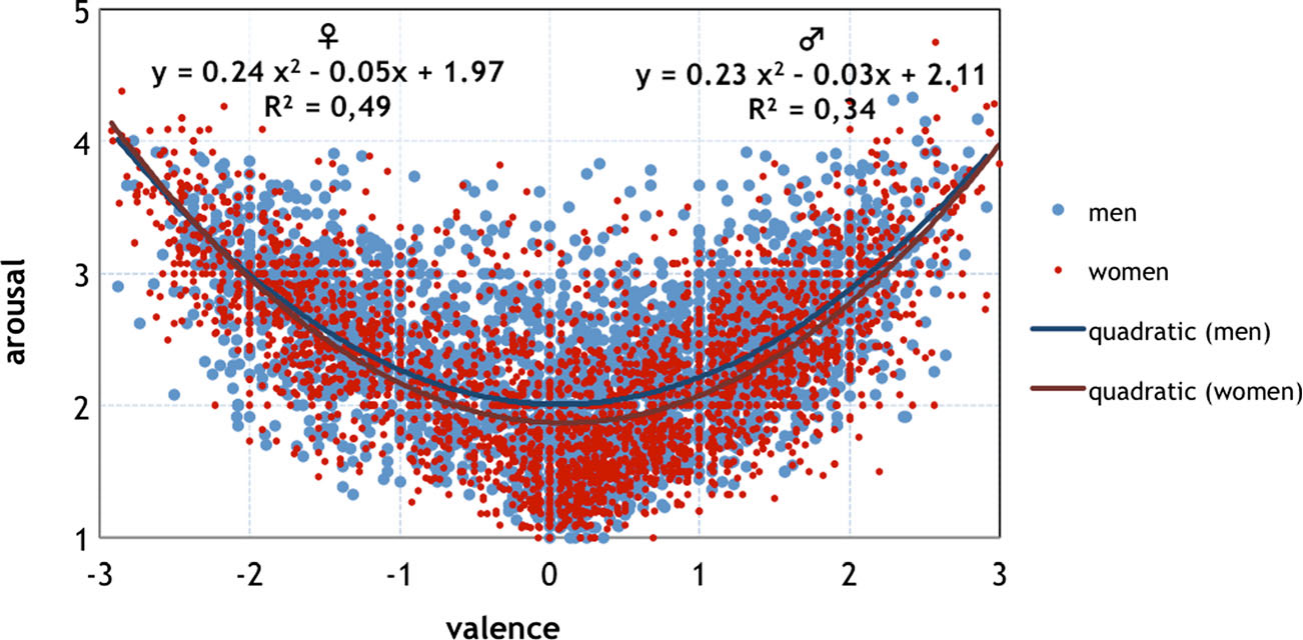
Mean ratings of the 2,902 Polish words for valence (*x*-axis) and arousal (*y*-axis), together with regression trend lines for men and women

To examine the issue of sex differences in the affective ratings, we analyzed the relationship between the ratings of valence and arousal for both males and females. The distributions of the affective ratings for all 2,902 words were similar for men and women, and both were classically U-shaped. Having conducted a regression analysis with valence as an independent variable and arousal as a dependent variable, we confirmed that this relationship between valence and arousal was best characterized by quadratic function for both males and females (*y* = 0.23*x* ^2^ – 0.03*x* + 2.11, *R* ^2^ = .34, and *y* = 0.24*x* ^2^ – 0.05*x* + 1.97, *R* ^2^ = .49, respectively; see Fig. 3). In other words, the quadratic relation between the two dimensions accounted for 34 % of the variance for males and 49 % for females, respectively. The ratings given by women were more extreme and formed a more pronounced U-shape than the ratings given by men. The linear model, in turn, accounted for only 0.1 % of the variance for men, and 1 % for women.

In order to obtain more precise conclusions about the impact of sex on the emotional evaluations, we conducted a multivariate repeated measures analysis of variance (MANOVA), taking words as the objects. We decided on this kind of analysis, because it has several advantages over multiple single ANOVAs. First, in an experiment examining several dependent variables, it helps to discover which factor is truly important. Additionally, MANOVA can protect against Type I errors that might occur for multiple ANOVAs conducted independently.

On the basis of the previous findings, we decided to investigate the possible effects of sex, for the groups of negative and positive words separately (Montefinese et al., 2014; Schmidtke et al., 2014; Soares et al., 2012). For this reason, we constructed a dummy variable transforming valence into negativity/positivity, with the following rule: The group of positive words included those with mean valence ratings above the middle of the rating scale (namely 0), whereas the group of negative words included those with mean valence ratings below 0.

Then we constructed a MANOVA model with a 2 × 2 × 3 mixed design: assuming Sex Category as a within-object factor, Negativity/Positivity Group as a between-object factor, and the mean ratings of words on the scales of valence, arousal, and imageability as the examined dependent variables.

It is important to note that such a MANOVA is only a rough approximation, given that the participants in the study assessed only portions of the whole range of words included in the NAWL, and that in contrast to usual procedures, sex was not considered a between-object factor.

In terms of the within-object effects, the analysis of variance showed a significant main effect of sex, *F*(3, 2860) = 33.49, *p* < .001, *η* ^2^ = .034. For the between-object effects, we found significant main effects of negativity/positivity for valence, *F*(1, 2862) = 6976.65, *p* < .001, *η* ^2^ = .071; arousal, *F*(1, 2862) = 145.55, *p* < .001, *η* ^2^ = .05; and imageability, *F*(1, 2862) = 100.23, *p* < .001, *η* ^2^ = .03. Also, the effect of the interaction of sex and the negativity/positivity of the words was significant, *F*(3, 2860) = 84.62, *p* < .001, *η* ^2^ = .082.

In order to better understand the nature of this interaction, we further analyzed the simple main effects of sex, for the groups of both negative and positive words. Pairwise comparisons showed that females rated valence significantly lower for the negative pictures (MD = –.23, *p* < .001), and significantly higher for the positive words (MD = .05, *p* < .001). Although females rated the arousal of negative words higher, this difference was not significant (MD = .01, *p* < .61), and females rated the arousal of positive words significantly lower than did males (MD = –.07, *p* < .001). The imageability of negative words was rated significantly lower by females (MD = –.09, *p* < .001), whereas the difference in the imageability ratings of positive words was not significant (MD = .01, *p* < .54).

Overall, the results we obtained showed that women probably were more radical in their assessments, with a tendency to assess words as being more extreme in valence (lower ratings for negative words and higher ratings for positive words) than men. As we previously reported, men showed a tendency to rate pleasant words as being more arousing than did women (Marchewka et al., 2014). Moreover, a significant effect of negativity/positivity revealed that negative words were assessed as being more arousing than positive words, confirming the already-reported findings regarding the relationship between valence and arousal.

To get the full image of the difference in the relation between affective variables in the groups of males and females, we conducted a multivariate curvilinear regression analysis. We treated arousal as a dependent variable and took valence and sex as predictors. The proposed model turned out to be significant (*F* = 861.98, *p* < .001). The *R* ^2^ value showed that the model explained 43 % of the variance of the dependent variable, namely arousal. The relation between the predictors and the dependent variable was significant for both the linear and quadratic components, including constant (*b*_0_ = 2.11, *p* < .001), valence (*b*_1_ = –.03, *p* < .001), sex (*b*_2_ = –.14, *p* < .001), valence squared (*b*_3_ = .23, *p* < .001), and the interaction of valence and sex (*b*_4_ = –.02, *p* < .05), but not the interaction of sex and valence squared (*b*_5_ = .01, *p* < .12). From Fig. 3, showing the curves estimated separately for the groups of males and females, it can be concluded that women rated neutral and positive words as being less arousing than did men, whereas they rated negative words as being more arousing than did men.

### Relations between the affective variables and the psycholinguistic variables

The results of the correlations computed for the affective dimensions and the objective and subjective psycholinguistic variables are presented in Table 3. This table shows that in the ratings of 2,902 Polish words, there was a weak but significant positive correlation between valence and lexical frequency. In other words, more frequently used words evoked more positive emotions. The arousal ratings were not related to frequency, yet they were related to word length, measured as the number of letters. This means that longer words were more arousing. Valence was positively related to imageability, and finally, imageability was negatively correlated with the number of letters. Negative words were the most arousing and most difficult to imagine. The longer the word, the more difficult it was to imagine.

**Table 3:** Correlation between valence and arousal ratings and the other psycholinguistic variables

	1	2	3	4	5
1. Valence	–	–.10^**^	.21^**^	–.04^*^	.06^**^
2. Arousal		–	.03	.09^**^	–.02
3. Imageability			–	–.29^**^	–.02
4. Number of letters				–	–.03
5. Frequency					–

^**^ *p* < .001, ^*^ *p* < .05

### Comparison with BAWL-R

Although the NAWL is a cultural adaptation of the BAWL-R, and thus contains many modifications aimed at adjusting the words to the Polish culture, the results showed that the affective ratings had similar distributions across the Polish and German cultures. Table 4 shows the correlations between the ratings from the German and Polish data sets.

**Table 4:** Correlation between the mean valence, arousal and imageability ratings from the NAWL and BAWL-R

Affective Data Sets	BAWL-R valence	BAWL-R arousal	BAWL-R imageability
NAWL valence	.85^**^	–.44^**^	.11^**^
NAWL arousal	–.11^**^	.55^**^	–.06^**^
NAWL imageability	.20^**^	–.13^**^	.65^**^

^**^ *p* < .001

**Fig. 4:**
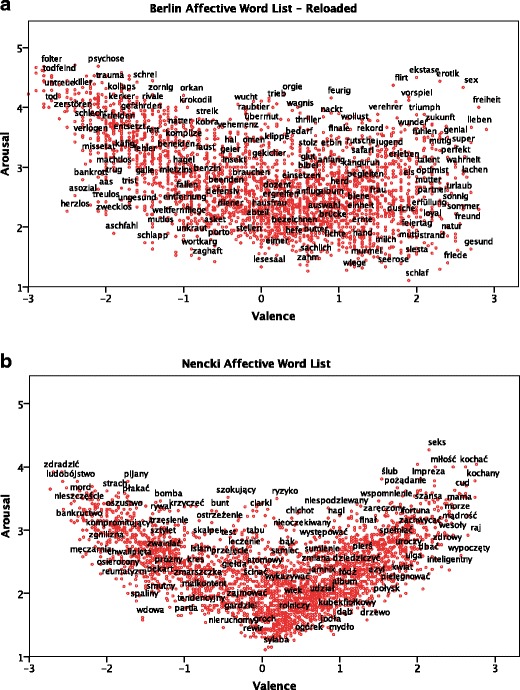
**a** Affective space of the Berlin Affective Word List–Reloaded in the dimensions of valence (*x*-axis) and arousal (*y*-axis), with exemplary words. **b** Affective space of the Nencki Affective Word List in the dimensions of valence (*x*-axis) and arousal (*y*-axis), with exemplary words

All three rating categories (valence, arousal, and imageability) in the two languages were significantly correlated, with the strongest correlation being for valence (*r* = .85 for valence, *r* = .55 for arousal and .65 for imageability). It should be emphasized that the upper bound for these correlations was set by reliability estimation, as described above. The obtained reliability measures were also highest for the valence scale, and lower for the arousal and imageability scales. Figure 4 shows the affective spaces of BAWL-R and the NAWL.

## Discussion

For the present study, we aimed at creating a Polish adaptation of the German BAWL-R (Võ et al., 2009) and collecting valence, arousal, and imageability rating for the 2,902 words it contains. Additionally, the words were estimated with regard to several objective psycholinguistic variables that are known to affect word processing—namely frequency, number of letters, and grammatical class.

Additionally, this study describes the characteristics of the word ratings in the database, by analyzing their distribution in affective space, possible gender-related differences in the valence, arousal, and imageability ratings, and the relationship between the affective and linguistic characteristics.

Our results concerning the distribution of ratings in the affective space are in line with studies conducted in other languages, which have shown that the relationship between valence and arousal is best described by a quadratic function (Eilola & Havelka, 2010; Redondo et al., 2007; Soares et al., 2012; Võ et al., 2009). This relation is characterized by symmetrically higher arousal values for emotionally valenced words, both positive and negative, than for neutral words. This is different from the asymmetrical relationship that has been demonstrated by many instruments that measure emotions from a bidimensional point of view, in which an increasing degree of negative valence is accompanied by an increase in emotional arousal, yet this relation is weaker for positive valence (Bradley & Lang, 1999; Redondo et al., 2007).

We examined the influence of gender on the affective ratings, since gender effects had been reported as significant in previous studies (Monnier & Syssau, 2014; Montefinese et al., 2014; Soares et al., 2012; Söderholm et al., 2013). First, we proposed a model of regression analysis with arousal as a dependent variable and valence and sex as predictors, which turned out to be significant and explained 45 % of the variance of our results. Subsequently, we compared the correlations between valence and arousal for both genders. In a regression curve estimation, we found that the quadratic function explained a larger part of the variance in the ratings of women (49 %) than of men (34 %), as had been reported in other studies (Monnier & Syssau, 2014). Our findings are in line with studies for the Portuguese adaptation of ANEW (Soares et al., 2012) and the Affective Norms for French Words (Monnier & Syssau, 2014), in that female participants rated words with more extreme valence values than did men. Having provided the affective norms separately for men and women will allow researchers to take into account sex differences in valence and arousal ratings when selecting their affective words. Although the origin of these gender differences in the affective ratings needs further investigation, we note the necessity for researchers interested in emotional words to take into account these interactions as potential sources of systematic error.

Our results are also in line with previous studies with respect to the idea that words’ affective characteristics are dependent on other psycholinguistic characteristics, the following of which we have provided: imageability, frequency, number of letters, and grammatical class. Specifically, we found that the more positive that words are on the valence scale, the more frequently they are used, which goes in line with previous studies (Montefinese et al., 2014; Warriner et al., 2013). This result might be linked to the so-called “linguistic positivity bias,” originally called “the Polyanna hypothesis,” meaning a tendency for positive words to be used more often than equally familiar negative words (Augustine, Mehl, & Larsen, 2011; Boucher & Osgood, 1969). Another previously reported result (Warriner et al., 2013) was that words easier to imagine were rated more positively. Longer words were more arousing, but also more difficult to imagine. As for the relations between the objective and subjective psycholinguistic indices, imageability correlated negatively with a word’s length measured in the number of letters. It has already been reported in previous studies (Quadflieg, Michel, Bukowski, & Samson, 2014) that the longer a word is, the more difficult it is to imagine. These findings point to the importance of controlling for psycholinguistic variables when investigating the effects of valence and arousal on cognitive processes.

As was already mentioned, the choice of rating scales was made according to the rating scales used in BAWL-R. The authors (Võ et al., 2009) reported that SAMs (Lang, 1980) were used for the arousal ratings as a 5-point instead of a 9-point scale. It is thus important to point out that in BAWL-R, and in NAWL accordingly, a rating of 5 on the arousal scale reflects the highest rating, whereas in most other studies using SAMs, that rating depicts a word that is neither arousing nor relaxing. Our understanding of the SAM procedure is also in line with that presented by the authors of BAWL-R (Võ et al., 2009), who noted that this procedure is not based on Lang’s (1980) theoretical and methodological approach to emotions, treating both emotional valence and emotional arousal as bipolar dimensions. We support the idea that valence is best represented by a bipolar dimension, with a negative pole on one side of the scale and a positive pole on the other, and neutral valences around the scale’s center. Nevertheless, we also agree with the difficulties resulting from “the absence of a clearly defined concept of ‘arousal’ ” (Ribeiro, Pompéia, & Bueno, 2005), and think that arousal can be explained better as a unipolar dimension, with a linear increase of positive values ranging from low to high arousal (values 1–5, respectively).

To reiterate, the results of our behavioral study creating the Nencki Affective Word List, a standardized affective verbal data set, are convergent with those of most studies conducted in this field of research. Therefore, the present database appears to be comparable with previously established norms.

### Description of the database

Table S1 attached in the supplementary materials to this article includes the ratings of the first 100 words in each of the two affective dimensions, along with the aforementioned psycholinguistic subjective ratings and objective indices. The database is organized in the following way:

- No.: number identifying each of the 2,902 words and corresponding to the original number in the BAWL-R (Võ et al., 2009)
- NAWL_word: Polish words in alphabetical order
- BAWL_word: original words from the BAWL-R (Võ et al., 2009)
- N_men, N_women, N_all: numbers of ratings obtained for each word from males, females, and the whole group

**Affective assessments**
- val_M_men: mean of the valence ratings for each word obtained in the group of men
- val_M_women: mean of the valence ratings for each word obtained in the group of women
- val_M_all: mean of the valence ratings for each word obtained in the whole group
- val_SD_men: standard deviation of the valence ratings for each word obtained in the group of men
- val_SD_women: standard deviation of the valence ratings for each word obtained in the group of women
- val_SD_all: standard deviation of the valence ratings for each word obtained in the whole group
- aro_M_men: mean of the arousal ratings for each word obtained in the group of men
- aro_M_women: mean of the arousal ratings for each word obtained in the group of women
- aro_M_all: mean of the arousal ratings for each word obtained in the whole group
- aro_SD_men: standard deviation of the arousal ratings for each word obtained in the group of men
- aro_SD_women: standard deviation of the arousal ratings for each word obtained in the group of women
- aro_SD_all: standard deviation of the arousal ratings for each word obtained in the whole group

**Psycholinguistic subjective indices**
- ima_M_men: mean of the imageability ratings for each word obtained in the group of men
- ima_M_women: mean of the imageability ratings for each word obtained in the group of women
- ima_M_all: mean of the imageability ratings for each word obtained in the whole group
- ima_SD_men: standard deviation of the imageability ratings for each word obtained in the group of men
- ima_SD_women: standard deviation of the imageability ratings for each word obtained in the group of women
- ima_SD_all: standard deviation of the imageability ratings for each word obtained in the whole group

**Psycholinguistic objective indices**
- Freq.: frequency, measured as the number of occurrences per million words (Pęzik, 2012)
- Let.: number of letters in each word
- Gram.: grammatical class, classified as nouns (N), verbs (V), and adjectives (A)
- SUBTLEX-PL: frequency and compound frequency measures based on movie subtitles (Mandera et al., 2014)

The NAWL is freely accessible to the scientific community for noncommercial use as supplementary material to this article.

### Electronic supplementary material

Below is the link to the electronic supplementary material.

ESM 1 (XLSX 615 kb)

## Appendix: instruction for word ratings

The original Polish instructions are presented, as well as the English translation.

### Instrukcja

Dziękujemy za Twoją zgodę na udział w badaniu.

Badanie dotyczy reakcji na czytane słowa. Przez około 60 minut będziesz czytać słowa wyświetlane na ekranie komputera. Twoim zadaniem będzie ocenić każde *z* 291 słów na trzech skalach. Po ocenieniu połowy *z* nich czeka Cię krótka przerwa.

Pierwsza skala to siedmiostopniowa skala znaku emocji, która określa, czy to słowo budzi w Tobie negatywne, czy pozytywne emocje

–3 = To słowo budzi we mnie bardzo negatywne emocje, 0 = To słowo nie budzi we mnie żadnych emocji, 3 = To słowo budzi we mnie bardzo pozytywne emocje

Druga skala to pięciostopniowa skala pobudzenia, jakie wzbudza w Tobie to, co opisuje dane słowo, mająca postać Manekina Samooceny

1 = Nie jestem pobudzony (jestem obojętny), 5 = Jestem pobudzony (np. jestem wzburzony lub podekscytowany)

Trzecią skalą jest siedmiostopniowa skala wyobrażalności, która określa, jak łatwo możesz sobie wyobrazić to, co opisuje dane słowo

1 = Bardzo trudno jest mi sobie wyobrazić to, co opisuje to słowo, 7 = Bardzo łatwo jest mi wyobrazić sobie to, co opisuje to słowo

Nie ma dobrych, ani złych odpowiedzi; odpowiadaj zgodnie *z* pierwszym skojarzeniem i staraj się wykorzystywać cały zakres skali ocen. W dowolnym momencie możesz wrócić do instrukcji, a potem kontynuować badanie.

Czytanie niektórych słów może Ci się wydać nieprzyjemne. Gdybyś poczuł, że nie chcesz kontynuować badania, możesz je przerwać w dowolnym momencie. W przypadku pytań, zwróć się do administratora badania.

### Instructions

Thank you for your consent to take part in the experiment.

The study concerns the reaction to the read words. For about 60 minutes you are going to read words shown on the computer screen. Your task will be to rate each of 291 words on three scales. Once you have rated half of them, there will be a short break.

The first scale is a 7-point scale of valence, which describes whether this word evokes negative or positive emotions

–3 = This word evokes very negative emotions in me, 0 = This word does not evoke any emotions in me, 3 = This word evokes very positive emotions in me

The second scale is a 5-point scale of arousal evoked by what this words describes, in the form of the Self-Assessment Manikin

1 = I am not aroused (I am neutral), 5 = I am aroused (for instance I am jittered or excited)

The third scale is a 7-point scale of imageability which describes how easily you can imagine what is described by this word

1 = It is very difficult for me to imagine what is described by this word, 7 = It is very easy for me to imagine what is described by this word

There are neither good nor bad answers; answer according to your first associations and try to use the whole range of the assessment scales. You can always come back to the instruction, and then continue the experiment.

Reading some of the words may seem unpleasant to you. Should you feel that you do not want to continue the experiment, you can stop it anytime you like. Once you have any questions, please address the administrator of the experiment.
